# High-Performance Advanced Composites in Multifunctional Material Design: State of the Art, Challenges, and Future Directions

**DOI:** 10.3390/ma17235997

**Published:** 2024-12-07

**Authors:** Sónia Simões

**Affiliations:** 1 Department of Metallurgical and Materials Engineering, Faculty of Engineering, University of Porto, Rua Doutor Roberto Frias, 4200-465 Porto, Portugal; ssimoes@fe.up.pt; Tel.: +351-220413113; 2 LAETA/INEGI-Institute of Science and Innovation in Mechanical and Industrial Engineering, Rua Doutor Roberto Frias, 4200-465 Porto, Portugal

**Keywords:** high-performance composites, advanced composites, multifunctional materials, smart composites, nanocomposites, additive manufacturing, material design

## Abstract

This review examines high-performance advanced composites (HPACs) for lightweight, high-strength, and multi-functional applications. Fiber-reinforced composites, particularly those utilizing carbon, glass, aramid, and nanofibers, are highlighted for their exceptional mechanical, thermal, and environmental properties. These materials enable diverse applications, including in the aerospace, automotive, energy, and defense sectors. In extreme conditions, matrix materials—polymers, metals, and ceramics—and advanced reinforcement materials must be carefully chosen to optimize performance and durability. Significant advancements in manufacturing techniques, such as automated and additive methods, have improved precision, reduced waste, and created highly customized and complex structures. Multifunctional composites integrating structural properties with energy storage and sensing capabilities are emerging as a breakthrough aligned with the trend toward smart material systems. Despite these advances, challenges such as recyclability, scalability, cost, and robust quality assurance remain. Addressing these issues will require the development of sustainable and bio-based composites, alongside efficient recycling solutions, to minimize their environmental impact and ensure long-term technological viability. The development of hybrid composites and nanocomposites to achieve multifunctionality while maintaining structural integrity will also be described.

## 1. Introduction

High-performance advanced composites (HPACs) are lightweight, high-strength, and durable. They overtake conventional materials, making them ideal for aerospace, automotive, and energy applications. The matrix may comprise polymers, metals, or ceramics, while reinforcements often use carbon, aramid, or glass fibers. The matrix and reinforcing material synergy give HPACs superior tensile, flexural, and environmental degradation resistance [1,2].

The significance of an HPAC lies in its ability to tailor properties for specific applications through carefully selecting matrix and reinforcement materials and their interface design. Recent innovations have expanded their capabilities beyond structural performance to include multifunctional properties such as self-healing, energy storage, and sensing, enabling a new generation of smart and adaptive materials. As demands for greater efficiency, sustainability, and innovation rise across industries, HPACs have become vital in addressing these challenges.

The importance of HPACs cannot be overstated, as they play a crucial role in modern engineering and technology. Their lightweight nature contributes to fuel efficiency in aerospace and automotive applications, where every gram saved can significantly reduce energy consumption and emissions. For instance, the aerospace industry has increasingly adopted advanced polymer-matrix composites (APMCs) due to their favorable strength-to-weight ratios, which enhance aircraft performance and reduce operational costs [3]. Furthermore, the ability of HPACs to withstand extreme temperatures and harsh environments makes them ideal for applications in nuclear reactors and other high-stress scenarios [4,5].

In addition to their mechanical advantages, HPACs offer significant versatility in manufacturing. Developing advanced manufacturing techniques, such as vacuum infusion, has produced complex composite structures with tailored properties [6]. These processes allow better control over the microstructure of composites, resulting in improved interfacial bonding between the matrix and the reinforcement [7]. Incorporating nanomaterials into the composite matrix enhances the mechanical and thermal properties of HPACs, enabling the design of materials that can effectively function under extreme conditions [8,9]. The environmental impact of HPACs is also a significant consideration in their development and application. As industries strive for sustainability, there is a growing emphasis on using bio-based and recyclable materials in composite manufacturing. Research shows that advanced biocomposites, which combine natural fibers with synthetic matrices, can achieve performance levels comparable to those of traditional composites while reducing dependence on petrochemical resources [10,11]. This shift toward greener materials addresses environmental concerns and opens new opportunities for innovation in composite technology.

The mechanical properties of HPACs are influenced by various factors, including the type of reinforcement used, the matrix material, and the manufacturing process [12]. For instance, using carbon fibers in epoxy matrices has resulted in composites with exceptional tensile and flexural strengths, making them suitable for high-performance applications [1,13]. Optimizing the interfacial properties between the fibers and the matrix is crucial to maximize load transfer efficiency, directly impacting the composite’s overall strength and durability [7]. In aerospace applications, the demand for HPACs is driven by the need for materials that can withstand high temperatures and mechanical stresses while maintaining structural integrity. Advanced composites such as carbon fiber-reinforced polymers (CFRPs) have been extensively used in aircraft components due to their lightweight and high-strength characteristics [1,3]. The ability to tailor the properties of these composites through careful material selection and processing techniques has led to significant advancements in aerospace engineering, enabling the design of more efficient and safer aircraft.

Furthermore, integrating advanced composites in civil engineering has revolutionized the construction industry. Using HPACs in structural applications allows for the creation of lighter and more durable structures, significantly reducing construction costs and improving sustainability [2,5]. Innovations such as developing liquid crystalline epoxy composites and using graphene-based nanomaterials are paving the way for the next generation of high-performance materials [8,14]. These advancements not only enhance the mechanical properties of composites but also introduce new functionalities, such as improved thermal conductivity and electrical insulation, which are critical for applications in electronics and energy storage [9,15].

**Figure 1:**
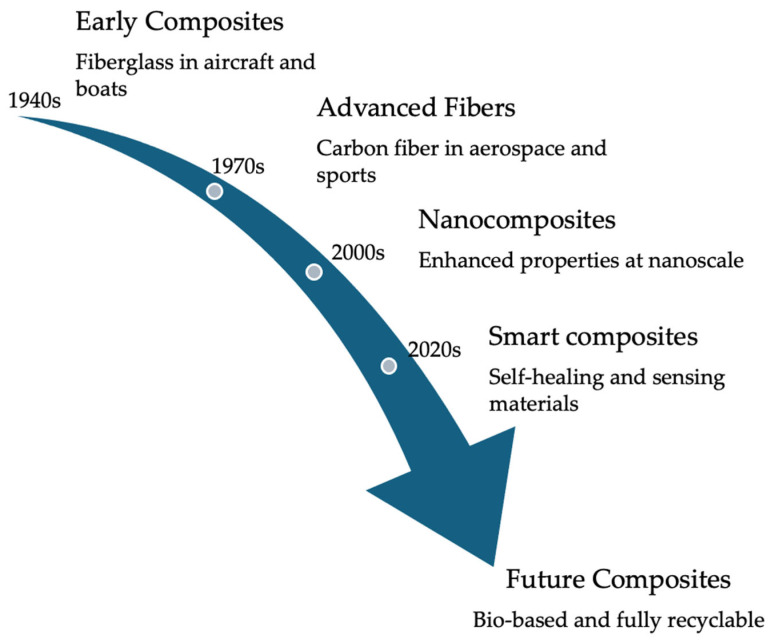
Evolution of composites [15].

The evolution of composite materials has enabled the production and development of modern advanced materials that can be used in the aerospace, automotive, and many other sectors, showcasing significant advantages. Figure 1 illustrates the evolution of composites. Significant advancements in composites have occurred since 1940 due to industrial and technological demands, particularly in the aerospace, automotive, and energy sectors. This evolution can be described in stages that reflect the development of materials, manufacturing techniques, and applications. For example, in the 1970s, the development and commercialization of carbon fibers represented a breakthrough. These materials offered high strength, stiffness, and low density, making them ideal for applications where weight reduction was critical, such as in the aerospace and defense industries. The use of fiber-reinforced polymers, such as fiberglass, expanded considerably. These composites were widely used in boats, aircraft, and structural components due to their corrosion resistance and high strength-to-weight ratio. In the 1980s, fiber-reinforced polymers, such as carbon fiber and fiberglass composites, were widely adopted in sports vehicles and architectural structures due to their durability and design flexibility. The 1990s saw the integration of advanced materials with the development of metal matrix composites (MMCs) and ceramic matrix composites (CMCs). Advances in surface treatments and interface engineering improved adhesion between fibers and the matrix, enhancing overall performance. A significant development in the 2000s was the development of nanocomposites. They were made by mixing carbon nanotubes and graphene into polymer matrices. This made it possible to make composites with great properties like high strength and good thermal and electrical conductivity. The 2010s focused on greater sustainability and developing composites with sensing and adaptive response capabilities, which were integrated into intelligent systems, such as materials used in aircraft and infrastructure. The continuous integration of nanotechnology, advanced manufacturing, and sustainable design promises a more versatile future for these materials [16].

This review paper aims to comprehensively analyze the current state of the art in high-performance advanced composites, detailing the advancements in material design, manufacturing processes, and performance enhancements. The scope covers various types of composites, including polymer matrix composites (PMCs), metal matrix composites (MMCs), ceramic matrix composites (CMCs), and hybrid systems.

## 2. Advanced Composites

Advanced composites comprise two or more constituent materials with significantly different physical or chemical properties. These composites typically include a matrix material, which can be polymeric, metallic, or ceramic, and a reinforcing phase, often in the form of carbon, glass, or aramid fibers. The unique properties of advanced composites include high specific strength, low density, and excellent fatigue resistance [17,18]. The significance of advanced composites lies in their ability to meet the demanding requirements of modern engineering applications. For instance, in the aerospace sector, advanced composites can reduce the weight of aircraft structures by approximately 25% to 30% compared to traditional metal structures, thereby enhancing fuel efficiency and overall performance [17]. This weight reduction is critical as it correlates with lower operational costs and reduced environmental impact due to decreased fuel consumption. In addition, advanced composites exhibit superior mechanical properties, such as high tensile strength and stiffness, essential for components subjected to extreme conditions [18]. The interface between the matrix and the reinforcing fibers plays a crucial role in determining the mechanical performance of advanced composites. The interfacial properties greatly impact how well stress is transferred between the matrix and fibers. This can greatly affect the composite material’s strength, durability, and thermal resistance. Therefore, optimizing the interface through various treatments or modifications is a vital area of research aimed at enhancing the performance of these materials [19]. In addition to their advantages in mechanical properties, advanced composites are increasingly being designed with environmental considerations. The development of hybrid composites and fiber-reinforced polymer (FRP) composites has gained prominence due to their enhanced properties and lower environmental impact than traditional materials. These composites are lightweight, strong, and exhibit good fatigue resistance and crashworthiness, making them suitable for applications in the automotive and energy sectors, where safety and sustainability are priorities [20]. Manufacturing processes for advanced composites have also evolved significantly, with resin transfer molding (RTM) and thermoforming becoming prevalent in producing complex shapes and structures [21]. The preforming stage in these processes is critical, as it sets the final shape of the composite parts, influencing their mechanical properties and performance in service [22]. Furthermore, advancements in curing methods, such as autoclave curing, have improved the efficiency and quality of composite fabrication, although these methods can be labor-intensive and costly [21].

The versatility of advanced composites extends to their applications in emerging fields such as biomedical engineering, where they are utilized in developing scaffolds, drug delivery systems, and tissue engineering materials. Adding nanostructured fillers to polymer matrices has created new ways to improve the functional properties of these composites, making them better able to meet the needs of advanced medical applications. In recent years, significant advancements have been made in smart composites thanks to the incorporation of cutting-edge materials and technologies that improve their functionality and applicability across various domains. Smart composites, characterized by their ability to respond to external stimuli, have emerged as critical components in aerospace, automotive, civil engineering, and biomedical applications. In addition to mechanical enhancements, the development of smart composites has also focused on improving their environmental sustainability. Incorporating bio-based materials, such as jute fibers, into composite formulations has garnered attention due to their renewable nature and lower environmental impact than traditional synthetic fibers [23]. The application of smart composites in structural health monitoring (SHM) has gained significant traction, particularly in civil infrastructure. Real-time structural integrity checks are now possible thanks to the development of piezoresistive smart-sensing composites [24,25]. These composites can detect changes in electrical resistance due to stress or damage. Such capabilities are essential for ensuring the safety and longevity of critical infrastructure, as they allow for timely maintenance and repair interventions. The integration of wireless detection systems further enhances the practicality of these smart composites, facilitating remote monitoring and data collection [24].

### 2.1. Classification of Composites

**Figure 2:**
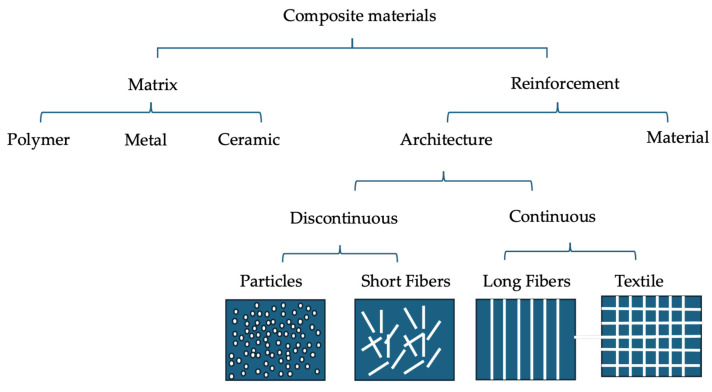
Classification of composites.

Composite materials are broadly classified based on the type of matrix material used. Each class has distinct properties that make it suitable for specific applications. The main categories are polymer matrix composites (PMC), metal matrix composites (MMC), ceramic matrix composites (CMC), and hybrid composites. Figure 2 shows a diagram of the classification of composites.

The following sections will present the most significant advances in composites.

#### 2.1.1. Polymer Matrix Composites (PMC)

Polymer matrix composites (PMCs) are the most widely used composites due to their versatility, ease of processing, and cost-effectiveness. PMCs consist of a polymer resin, such as epoxy, polyester, or thermoplastic, reinforced, for instance, with carbon, glass, or aramid fibers. The content of fibers incorporated into the matrix plays a critical role in determining the performance characteristics of composite materials. Fibers act as the primary reinforcement within the composite, providing mechanical strength, stiffness, and resistance to various stresses. At the same time, the matrix serves as the binder that holds the fibers together and facilitates load transfer. Fiber content is typically measured as a weight fraction (Wf) or volume fraction (Vf), typically between 20% and 70%. Adjusting the fibers’ type, orientation, and distribution allows for the tailoring of composites to meet precise performance requirements. The lightweight nature of polymer matrices, combined with the high strength and stiffness of the reinforcement, results in materials with an excellent strength-to-weight ratio, making them ideal for aerospace, automotive, and oilfield applications [26,27].

**Figure 3:**
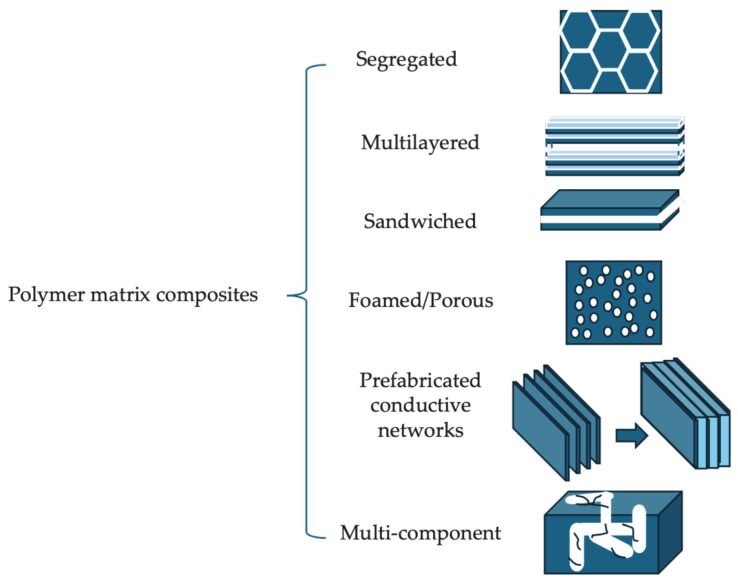
Polymer matrix composite structure designs.

Recent developments in PMCs focus on enhancing their multifunctional properties, such as electrical conductivity and self-healing capabilities, by incorporating nanomaterials like carbon nanotubes and graphene. Additionally, research efforts aim to improve their environmental sustainability, with bio-based polymers and natural fibers being explored as alternatives to traditional petroleum-based materials. Advances in PMCs have significantly transformed various industries, particularly in electronics, aerospace, and biomedical applications. These advancements are primarily driven by incorporating innovative fillers and developing novel processing techniques that enhance PMCs’ mechanical, thermal, and electrical properties [28,29]. Figure 3 shows the designs offer diverse strategies for balancing conductivity, mechanical strength, and weight, providing a comprehensive overview of the potential applications of these materials in various industries.

One of the notable advancements in PMCs is the enhancement of thermal conductivity, which is crucial for applications in high-performance electronics and 5G technology. Recent studies have demonstrated that incorporating thermally conductive fillers, such as graphene and metal oxides, can significantly improve the heat dissipation capabilities of polymer composites. For instance, Yu et al. [30] highlighted that using thermally conductive fillers in PMCs has been pivotal in improving the heat dissipation capacity of electronic devices, thereby enhancing their performance and reliability. Similarly, Zhu et al. [31] reviewed the mechanisms influencing thermal conductivity in PMCs, emphasizing the importance of filler distribution and the formation of thermal networks within the composite matrix. These results underscore the critical role of material selection and processing techniques in achieving desired thermal properties.

Moreover, the mechanical properties of PMCs have also seen substantial improvements through hybridization techniques. Nayak et al. [32] explored the mechanical performance of hybrid composites, demonstrating that combining different fibers can enhance load-bearing capabilities and reduce manufacturing costs. This hybrid approach optimizes composite properties, making them more suitable for various applications. Additionally, using natural fibers as reinforcement in PMCs has gained traction due to their sustainability and cost-effectiveness. Hejna et al. [33] emphasized the compatibility of wood-polymer composites in various industrial applications. Wood-polymer composites have revolutionized the materials landscape with their unique blend of sustainability, versatility, and performance. The versatility of wood-polymer composites opens possibilities for innovative designs and applications across different sectors, such as construction, automotive, and furniture manufacturing. The automotive sector has also embraced wood-polymer composites for interior components, providing a lightweight and eco-friendly option for car manufacturers looking to reduce carbon emissions. Researchers continue to explore new ways to enhance the performance of wood-polymer composites through advancements in material science and technology, ensuring they meet the demands of various industrial applications.

**Figure 4:**
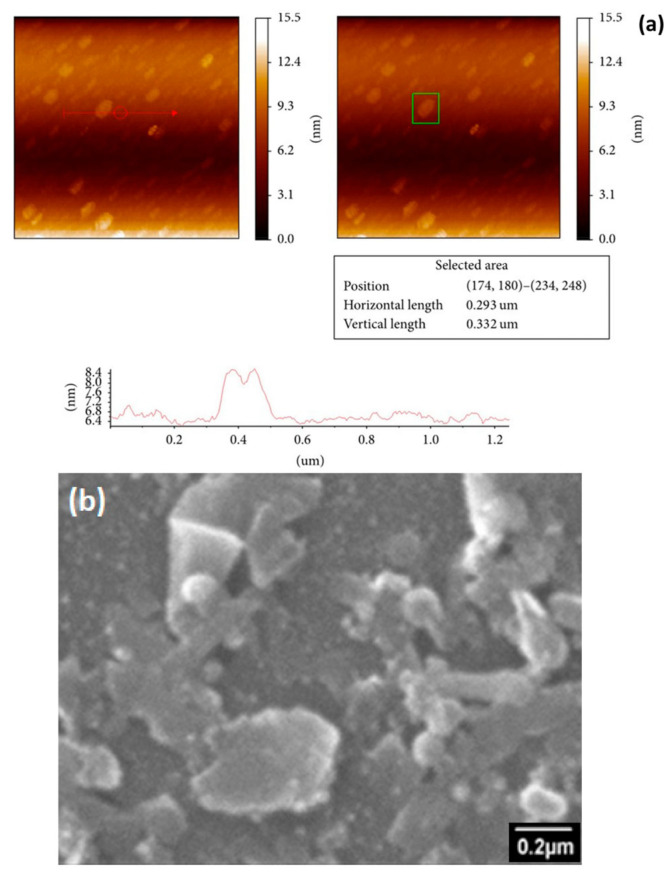
(**a**) Atomic force microscopy (AFM) image along with dimensional measurements (red line and green marked) showing the length (L) and thickness (t) of MoS₂ nanosheets; (**b**) scanning electron microscopy (SEM) image of exfoliated MoS_2_ [34].

Recent advancements in electromagnetic interference (EMI) shielding have focused on developing PMCs with enhanced dielectric properties [34,35]. Saboor et al. [34] reported synthesizing hybrid polymer composites that exhibit high dielectric behavior, which is essential for effective EMI shielding in electronic devices. Incorporating polyaniline and metal oxides has improved PMCs’ electrical conductivity and dielectric permittivity, making them suitable for applications requiring effective EMI protection. The authors demonstrated that molybdenite (MoS₂) sheets exfoliated in solution and polyaniline (PANI) nanoparticles are dispersed in a polystyrene (PS) matrix can produce hybrid polymer composites with high dielectric and electromagnetic interference (EMI) shielding behavior. Figure 4 shows the atomic force microscopy (AFM) and scanning electron microscopy (SEM) images of the molybdenum disulfide (MoS_2_) nanosheets. When PANI and MoS_2_ are incorporated into the polystyrene matrix, a morphology with separate phases is formed. As the concentration of MoS_2_ nanoparticles increases in the PS/PANI polymer blend (5% by weight), an interconnected network is created, which results in high electrical conductivity and dielectric behavior, making these materials promising candidates for EMI shielding applications. An increase in dielectric constant and dielectric loss of up to four and five orders of magnitude is observed at the maximum concentration of 1% by weight of MoS_2_ in the PS/PANI-5 blend at 100 Hz. The enhanced dielectric characteristics of the PS/PANI/MoS_2_ composites are evaluated theoretically to estimate the EMI shielding effectiveness in the 100 Hz to 5 MHz frequency range. The higher dielectric constant and loss recorded for the PS/PANI-5%/MoS_2_-1% composite is responsible for an estimated shielding effectiveness of around 92 dB at 100 Hz. The increase in dielectric behavior and shielding efficiency is probably due to the more significant accumulation of charged dipoles at the insulator-conductor interface.

Also, Iqbal et al.’s work [36] on MXene-based composites shows how the structure can be changed to improve EMI shielding. This shows that more research is needed in this area. The environmental impact of PMCs has also prompted researchers to explore eco-friendly alternatives and recycling methods. The work by Liao et al. [37] on recycling aramid fiber waste into new composite materials illustrates the potential for sustainable practices in PMC production. Additionally, the eco-design principles discussed by Lazăr [38] emphasize the importance of sustainability in PMCs’ design and manufacturing processes, advocating for a holistic approach to reduce environmental footprints.

#### 2.1.2. Metal Matrix Composites (MMC)

MMCs have garnered significant attention in recent years due to their exceptional mechanical properties and versatility in various applications, particularly in aerospace, automotive, and structural engineering. Advances in the field have been driven by incorporating reinforcement materials, such as ceramics, carbon allotropes, and other nano-sized particles, which enhance the performance characteristics of the metal matrix. One of the primary advantages of MMCs is their ability to combine the beneficial properties of metals with those of reinforcing materials. For instance, aluminum-based composites reinforced with silicon carbide (SiC), titanium carbide (TiC), and carbon nanotubes (CNTs) exhibit improved strength, stiffness, and wear resistance compared to their unreinforced counterparts [39,40,41,42]. The use of nano-sized reinforcements, such as graphene and carbon nanotubes, has been encouraging, as these materials can significantly enhance the mechanical properties of the matrix due to their high specific strength and excellent thermal and electrical conductivity [42,43].

**Figure 5:**
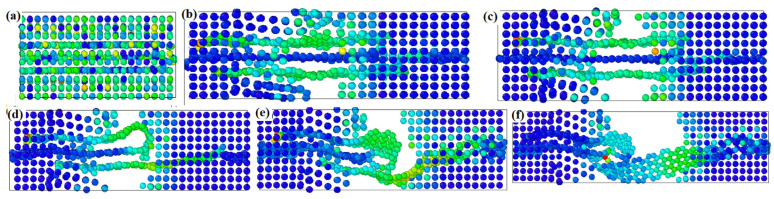
Stress distribution of three-layer graphene/aluminum composite during tensile failure. (**a**–**f**) illustrates the sequence of events, from the initial formation of cracks in the matrix to the eventual failure of the material during stretching [42].

Huang et al. [42] show that, in the case of an aluminum matrix reinforced with single-layer graphene, the simulated stretching process does not cause the graphene to break as the deformation increases. Instead, a shearing behavior occurs between the graphene and the aluminum matrix, causing the graphene to be “pulled” from the aluminum matrix. In the parallel elongation direction, tensile stress tends to increase as the proportion of graphene area increases. In the vertical stretching direction, the tensile stress tends to decrease as the percentage of graphene area increases. In the direction parallel to elongation, the tensile stress of the system tends to decrease as the angle between the graphene and the elongation direction increases. Figure 5 shows the stress distribution of a three-layer graphene/aluminum composite during tensile failure. Studying the impact of different graphene distributions in the aluminum matrix on the mechanical properties of composites is fundamental for developing metal matrix/graphene composites with high strength.

**Figure 6:**
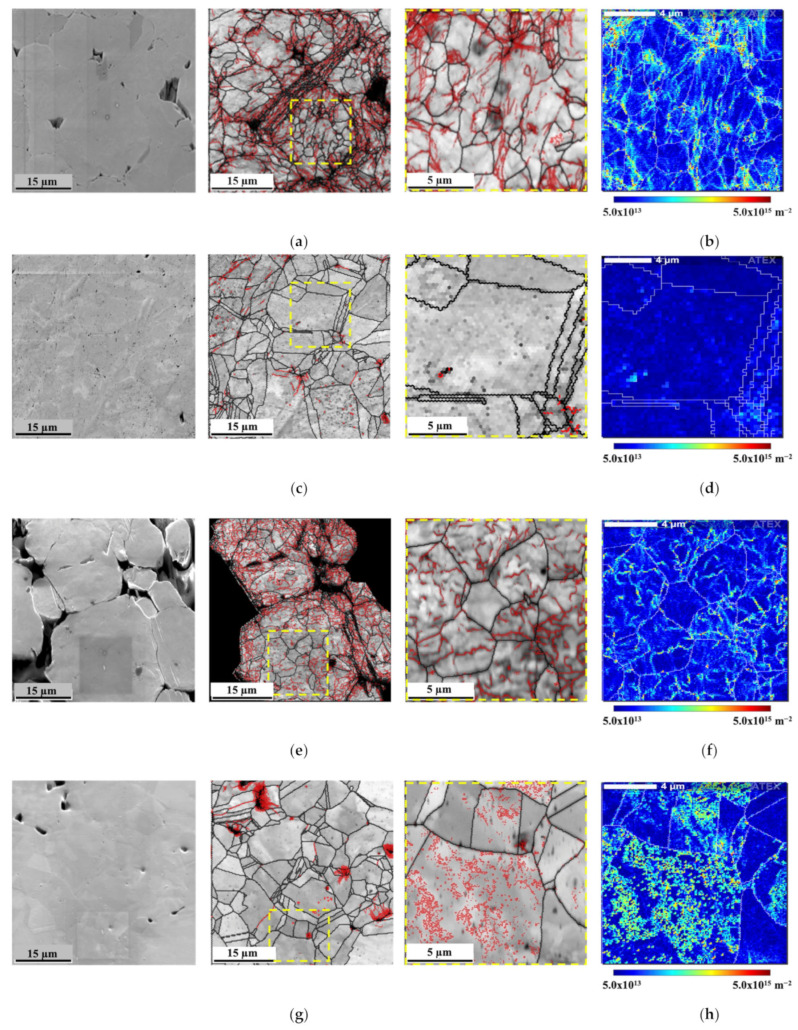
SEM images and IQ maps with high- and low-angle boundaries (black and red, respectively) delimited and geometrically necessary dislocation (GND) density maps for (**a**,**b**) Ni green compact, (**c**,**d**) sintered Ni, (**e**,**f**) Ni-CNT green compact, and (**g**,**h**) Ni-CNT sintered samples [44].

The development of aluminum-potassium feldspar composites has also shown potential for automotive applications, demonstrating favorable mechanical properties while maintaining a density like that of aluminum alloys [43]. The manufacturing processes for MMCs have evolved significantly, with techniques such as powder metallurgy, stir casting, and additive manufacturing being employed to achieve the desired material properties and microstructures. For instance, powder metallurgy allows for the precise control of the reinforcement distribution within the matrix, which is crucial for optimizing mechanical performance [44,45]. For example, Carneiro et al. [44] show that a Ni matrix reinforced with CNTs shows significant improvements in mechanical properties, which can be associated with microstructural changes related to the addition of the reinforcement. The authors characterized the nanocomposites by backscattered electron diffraction (EBSD), which shows that the dispersion/mixing and pressing processes cause plastic deformation in the powders received. The dislocation structures generated in these initial steps are partially eliminated during sintering due to the activation of recovery and recrystallization mechanisms. However, the presence of CNTs in the matrix significantly impacts the annihilation of dislocation, thus reducing the recovery of these structures. Figure 6 shows the EBSD maps of the matrix and nanocomposites pressed and sintered under the same conditions, showing the microstructural changes induced by the presence of the reinforcement.

Additionally, advancements in hybrid metal matrix composites (HMMCs) have enabled the combination of multiple reinforcement types, further enhancing their mechanical properties and broadening the range of applications [46,47]. Moreover, the thermal properties of MMCs are of great interest, particularly in applications where thermal management is critical. The matrix and reinforcement materials’ thermal expansion coefficients (CTEs) must be carefully matched to minimize internal stresses during processing and service [42,48]. This aspect is particularly relevant when considering using MMCs in high-temperature environments, where maintaining structural integrity is paramount. The environmental impact of MMCs is also a growing concern, leading to research focused on sustainable materials and processes. For example, using waste materials, such as groundnut shells, as reinforcement in aluminum composites has been explored to reduce environmental pollution while enhancing material properties [48,49]. This trend reflects a broader movement towards developing eco-friendly materials without compromising performance.

#### 2.1.3. Ceramic Matrix Composites (CMC)

Ceramic matrix composites (CMCs) have emerged as a significant class of materials, particularly in high-performance applications such as in the aerospace, automotive, and defense industries. A fiber-reinforced ceramic matrix improves the mechanical properties of these materials. This keeps the benefits of ceramics, like being stable at high temperatures and corrosion resistance. Recent advancements in CMCs have focused on improving their performance under extreme conditions, addressing challenges such as brittleness and oxidation.

One of the primary benefits of CMCs is their ability to withstand high temperatures, making them suitable for applications in aerospace propulsion systems. For instance, fiber-reinforced ceramic matrix composites (FRCMCs) have been developed to offer superior high-temperature stability compared to traditional superalloys, which are reaching their operational limits in modern aerospace vehicles [50]. Incorporating fibers, such as silicon carbide (SiC) and carbon fibers, into the ceramic matrix enhances toughness and contributes to weight reduction, which is critical for aerospace applications [51,52,53]. Furthermore, the thermal stability of advanced structural ceramics can lead to significant fuel savings in aircraft engines, highlighting their potential economic benefits [54].

**Figure 7:**
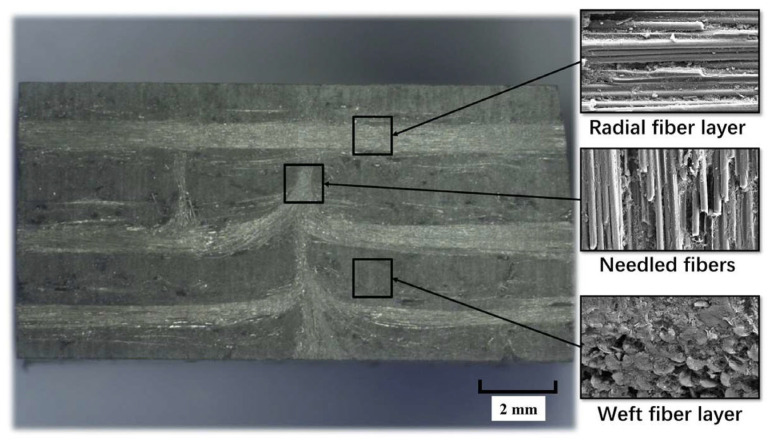
Microscopic morphology of the cross-section of 2.5D Cf/SiCs [51].

Wen et al. [51] investigated the process and performance of micromachining 2.5D Cf/SiC and 2.5D SiCf/SiC composites in detail. Figure 7 shows the morphology of the composites. Single-factor micromachining experiments were carried out using SiC ceramics as a reference. The differences in the material removal process, surface microstructure, surface roughness, and grinding force of the three materials under the same grinding parameters were comparatively analyzed. The results indicate that crack propagation is intense during the micro-grinding process of SiC ceramics. The machined surface shows irregularities, with cavity-shaped defects and high surface roughness. However, reinforcing fibers and interfaces in both composites helps to stop cracks from spreading or changing the direction in which they grow. This makes the surfaces smooth and flat after grinding, with few flaws and low Ra roughness. In addition, the orientation of the fibers affects the grinding process in both composites. The crack propagation paths and fracture positions of the fibers in the web and radial fiber layers are different, leading to different grinding defects.

Despite their advantages, CMCs face challenges, particularly in oxidative environments. Research indicates that CMCs can be prone to oxidation and surface recession when exposed to high water vapor content, which limits their applicability in specific high-temperature environments [52]. This means that more research needs to be conducted on protective coatings and other fiber materials that can make CMCs more resistant to oxidation and increase their usefulness [55]. Recent innovations have also explored the integration of nanomaterials, such as graphene and carbon nanotubes, into CMCs. These materials can significantly improve the mechanical properties of the composites, including hardness and wear resistance [56,57]. Using graphene nanoplatelets has been a good idea because they can improve the thermal and electrical properties of the ceramic matrix. This means they can be used for many things, like blocking electromagnetic interference and storing energy [58]. New processing methods, like powder injection molding and slurry infiltration, have made it easier to make CMCs that are more structurally stable and perform better [59,60]. These advancements have also allowed for the development of more complex and intricate CMC components, expanding their potential applications in various industries. Furthermore, these components’ increased durability and heat resistance make them ideal for high-temperature environments.

#### 2.1.4. Hybrid Composites

Hybrid composites incorporate two or more different types of fibers or matrices to achieve a balance of properties that single-fiber composites cannot provide. For example, hybrid composites can combine carbon fibers for strength and stiffness with glass fibers for cost-effectiveness and impact resistance, resulting in materials with a well-rounded performance profile. These composites are increasingly being explored for applications requiring a combination of mechanical, thermal, and functional properties. These materials allow designers to tailor composites for specific applications by strategically combining different fibers and matrices, offering enhanced performance, durability, and versatility.

This innovative approach allows for the exploitation of the unique properties of each constituent material, leading to composites that exhibit superior mechanical, thermal, and ecological properties compared to traditional single-material composites. The hybridization process enhances performance and addresses the limitations associated with individual materials, making hybrid composites a focal point of contemporary research and application. Table 1 shows an overview of hybrid composite design and the application and advantages of these materials.

One of the primary motivations for developing hybrid composites is to achieve a balance between strength, weight, and cost-effectiveness. For instance, studies have shown that combining natural fibers with synthetic fibers, such as glass or carbon, can significantly improve the mechanical properties of the resulting composite materials. This synergy allows for creating strong, lightweight, and environmentally friendly materials, as they can incorporate renewable resources [70,71,72]. Research has demonstrated that hybrid composites can outperform their non-hybrid counterparts in tensile strength and impact resistance, making them suitable for demanding applications in the automotive and aerospace industries [73,74]. The mechanical performance of hybrid composites can be tailored by carefully selecting fiber types and matrix materials. For example, incorporating natural fibers, such as kenaf or pineapple leaf fibers, into a polypropylene matrix has enhanced its mechanical properties and water absorption characteristics [74,75].

Additionally, new processing methods like hot press molding and 3D weaving have made it easier to create hybrid composites with complex shapes and better interfacial bonding, which are important for improving overall performance [76]. The ability to manipulate fiber orientation and matrix composition further allows for optimizing specific properties, such as thermal stability and acoustic insulation [77,78]. Moreover, the versatility of hybrid composites extends to their applications in various fields, including construction, automotive, and aerospace. Integrating high-strength fibers, such as Kevlar or carbon, with natural fibers in hybrid composites has benefited applications requiring high-impact resistance and low weight [68]. Recent studies have highlighted the potential of hybrid composites in ballistic applications, where combining different fiber types can enhance the material’s ability to withstand extreme conditions [79]. The eco-friendly nature of natural fiber-reinforced hybrid composites aligns with the growing demand for sustainable materials in modern engineering practices [73,80].

## 3. Advanced High-Performance Composites

Recent advances in advanced high-performance composites (AHPCs) have significantly transformed the materials science landscape, leading to innovative applications across various industries. These composites, characterized by their exceptional mechanical properties, lightweight nature, and versatility, are increasingly utilized in the aerospace, automotive, and construction sectors. The integration of novel materials, advanced manufacturing techniques, and sustainable practices has propelled the development of AHPCs, making them a focal point of research and application. One of the notable advancements in AHPCs is the development of bio-based thermosetting resins, which are gaining traction due to their sustainable nature and high performance. Recent studies have highlighted the potential of using renewable feedstocks to create thermosetting composites that meet the stringent requirements of aerospace and marine applications [81,82]. These bio-based materials reduce reliance on petroleum-based products and exhibit mechanical properties comparable to traditional composites, thus promoting a more sustainable approach to materials engineering.

Furthermore, using bio-based thermosetting resins reduces carbon emissions and overall environmental impact. This shift towards sustainable materials in AHPCs aligns with the growing industry’s emphasis on eco-friendly practices. As research and development in this field progresses, we expect to see even more innovative and environmentally conscious solutions emerge, further revolutionizing how high-performance composites are manufactured and utilized.

**Figure 8:**
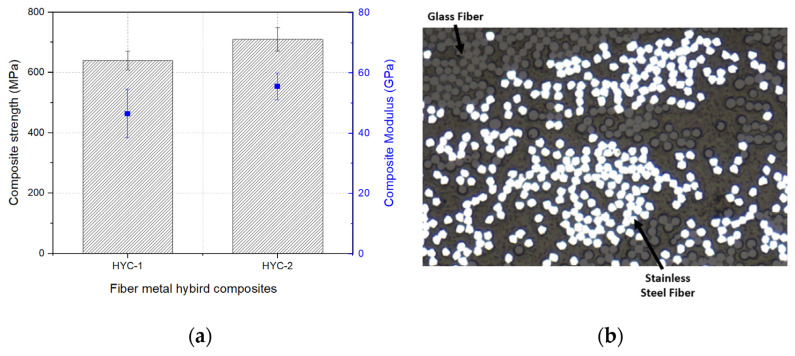
GF/stainless-steel/PA6 hybrid composites: (**a**) tensile properties and (**b**) cross-sectional image of hybrid composite [84].

Due to the growing demand for sustainable manufacturing and waste management techniques, recycling composite materials has also become a crucial area of research. The state-of-the-art recycling technologies for composites have been explored, particularly in response to the growing sociotechnical pressures for sustainable solutions. For instance, decommissioning wind turbine blades and aircraft has prompted the development of efficient recycling methods to reclaim valuable materials from end-of-life composites, thereby contributing to a circular economy [83]. This focus on recycling not only addresses environmental concerns but also enhances the economic viability of composite materials. In multifunctional composites, recent innovations have introduced hybrid materials that combine high-performance fibers with metals to achieve enhanced mechanical properties and functional characteristics. For example, developing glass/stainless steel/polyamide commingled yarns has demonstrated improved impact resistance and energy absorption capabilities, making them suitable for demanding applications. This hybridization approach not only enhances the mechanical performance of the composites but also provides additional benefits, such as increased electrical conductivity and thermal resistance [84]. Figure 8 shows the results of these hybrid composites. The results demonstrate a significant improvement in mechanical strength and conductivity compared to traditional composites.

Another significant advancement is the incorporation of shear-thickening gels (STGs) into high-performance fiber-reinforced composites. STGs exhibit unique properties that allow them to transition from liquid to solid under stress, providing exceptional energy absorption capabilities [85]. This characteristic is particularly advantageous in protective applications, such as body armor, where the ability to absorb and dissipate impact energy is crucial [86]. Integrating STGs into composite materials represents a promising direction for developing advanced protective gear that can withstand high-velocity impacts while maintaining flexibility. Aadditionally, bioinspired design principles have led to the creation of multilayered cellular composites that mimic the mechanical properties of natural materials, such as nacre and cuttlebone. These composites exhibit enhanced energy absorption and shape recovery capabilities, making them ideal for applications requiring high-impact resistance. The hierarchical structure of these materials allows for efficient load distribution and crack propagation control, which are essential for improving the durability and performance of composite structures [87]. The exploration of novel manufacturing techniques, such as additive manufacturing and laser lithography, has also contributed to the advancement of AHPCs. These methods enable the production of complex geometries and tailored microstructures that enhance the performance of composite materials [88,89]. For instance, the ability to create intricate designs through additive manufacturing allows for optimizing material properties and reducing waste, aligning with sustainable manufacturing principles. These advancements in material design have the potential to revolutionize various industries and improve overall product efficiency.

### 3.1. Materials Selection and Design Strategies

The selection and design strategies for advanced composite materials are critical for optimizing their performance in various applications. The strategic selection of materials and design methodologies is essential to harness these benefits effectively. One of the primary considerations in materials selection is the combination of high-strength fibers with appropriate matrix materials. For instance, carbon or aramid fibers combined with epoxy resins can yield composites that exhibit an optimal balance of mechanical, thermal, and chemical properties [2]. This combination allows for tailored properties that meet specific application requirements, such as enhanced durability and resistance to environmental degradation [90]. Moreover, integrating advanced computational tools and multiscale modeling techniques has revolutionized the design process, enabling engineers to predict performance more accurately and efficiently [2]. This has resulted in significant advancements in the field of engineering.

Another significant aspect of materials selection is using multi-criteria decision-making (MCDM) tools, which facilitate the evaluation of various material options based on multiple performance criteria. These tools help designers navigate the complexities of selecting materials that meet diverse mechanical and physical property requirements [91]. For example, the analytic hierarchy process (AHP) has effectively prioritized material selection in automotive applications, ensuring that the chosen materials align with performance specifications while considering sustainability [92,93]. The hybridization of natural and synthetic fibers is also a growing trend, as it can enhance toughness and provide a more sustainable alternative to traditional composites [80]. The design strategies for advanced composites also involve considerations of anisotropic properties, which can be tailored through fiber orientation and layering techniques. This customization allows for production composites that respond differently under various loading conditions, enhancing their functional performance [94]. However, this increased flexibility comes with added complexity, necessitating sophisticated design methodologies that can accommodate the intricate interactions between material properties and structural requirements [95] to ensure optimal performance and safety of the final product.

**Figure 9:**
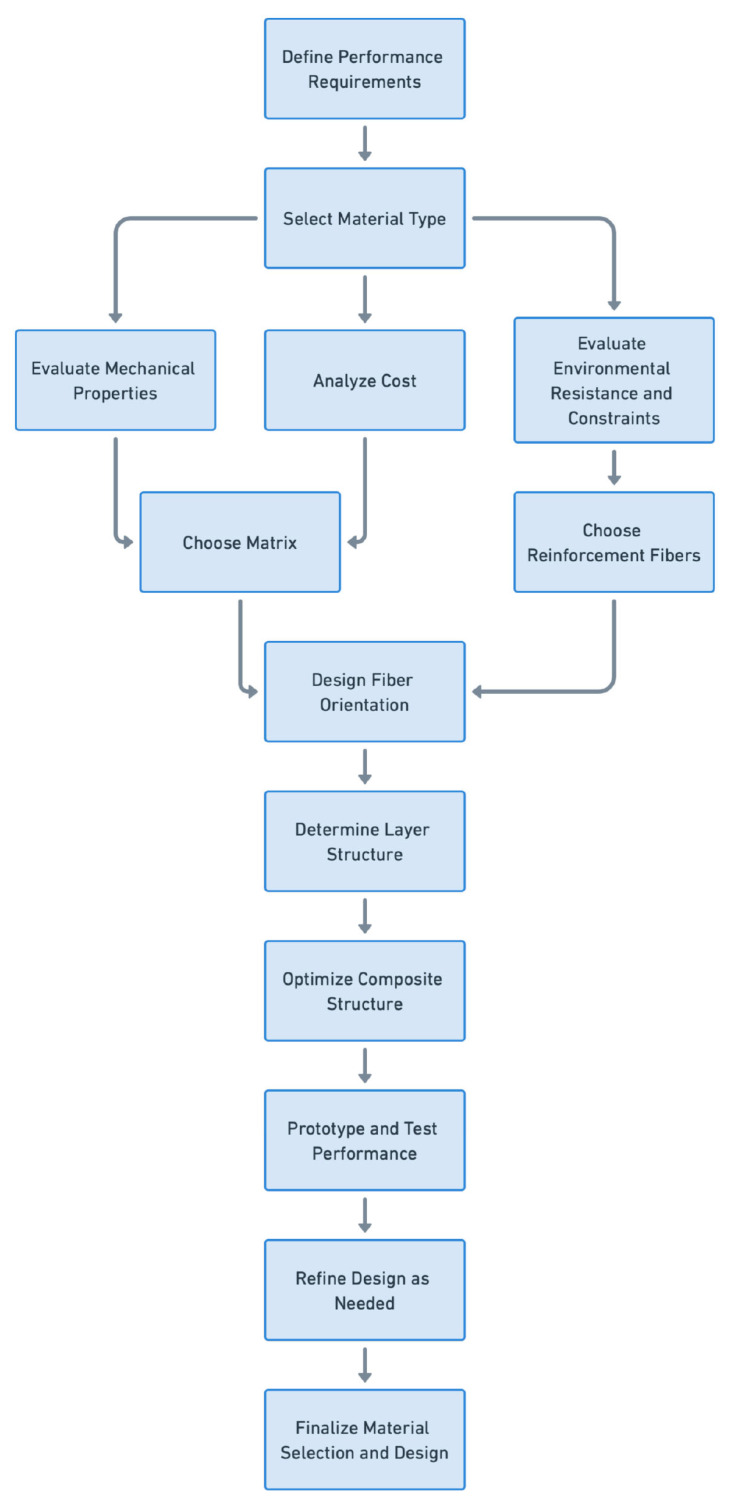
Flowchart representing the materials selection and optimization for advanced high-performance composites.

Furthermore, the advent of eco-friendly composites, which utilize biodegradable materials and natural fibers, is reshaping the landscape of composite design. These materials offer reduce environmental impact and maintain competitive mechanical properties, making them suitable for various applications [96]. Integrating biocomposites into product design reflects a broader trend toward sustainability in engineering practices, highlighting the importance of selecting materials that align with environmental considerations. Figure 9 shows the flowchart representing the selection and optimization of the material for advanced high-performance composites. This flowchart illustrates a systematic approach to composite material design, ensuring all factors like performance, environmental conditions, cost, and structural requirements are addressed to create a robust and optimized composite structure. One branch of the flow evaluates the mechanical properties essential for the application, such as strength, stiffness, and toughness. The design of the composite structure is optimized, integrating all previous decisions to maximize performance within constraints. The other branch optimizes the material layout and processing conditions to improve manufacturability and reduce costs.

#### 3.1.1. Reinforcement

**Figure 10:**
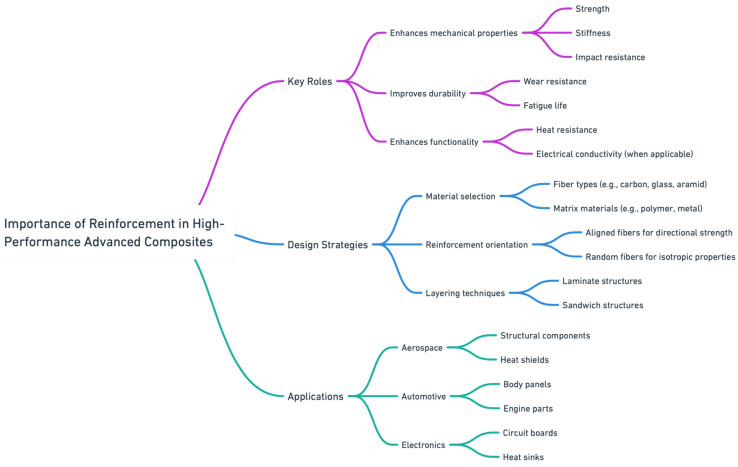
Flowchart representing the materials selection and optimization for advanced high-performance composites.

The importance of reinforcement in the materials selection and design strategies of high-performance advanced composites cannot be overstated. Figure 10 shows a flowchart representing the materials selection and optimization for advanced high-performance composites. The reinforcement is critical in enhancing composite materials’ mechanical properties, durability, and functionality, which are increasingly utilized in various high-tech applications, including aerospace, automotive, and electronics. Carbon fibers are widely used in high-performance composites due to their excellent strength-to-weight ratio, high stiffness, and thermal stability. They are commonly used in aerospace, automotive, and sporting applications where lightweight materials with superior mechanical properties are critical. Carbon fibers also offer good fatigue resistance, making them ideal for long-term, load-bearing applications.

Reinforcement mechanisms in advanced composites often involve incorporating various materials, such as nanoparticles, fibers, and other fillers, which significantly improve the mechanical performance of the base matrix. For instance, using carbon nanotubes and graphene as reinforcements has enhanced the strength and toughness of polymer matrices, leading to composites that exhibit superior mechanical properties compared to traditional materials [97,98]. These developments are crucial because the positioning and distribution of these reinforcing elements within the matrix frequently determine the mechanical performance of composites. Controlling the three-dimensional orientation of reinforcing particles has been shown in recent studies to make composites with specific mechanical properties. These composites can have complex structures like those found in natural materials like bone and shells [99,100].

Moreover, the design strategies for high-performance composites are evolving by integrating machine learning (ML) techniques. These approaches allow for exploring vast compositional spaces and optimizing material properties based on learned structure-property relationships. For example, ML has been successfully applied to generate high-performing designs for graphene nanocomposites, demonstrating its potential to streamline the design process and enhance the performance of advanced composites [101]. This computational approach complements traditional experimental methods, enabling researchers to predict the outcomes of various reinforcement strategies before physical trials. The hierarchical design principles observed in biological composites also inspire the development of advanced materials. By simulating the heterogeneous structures found in nature, researchers can create composites that exhibit exceptional strength, toughness, and flexibility combinations. For instance, studies have shown that composites designed with layered architectures can achieve remarkable mechanical properties, broadening the scope of applications for these materials [98,100]. This biomimetic approach enhances performance and promotes sustainability using eco-friendly materials and processes. In addition to mechanical enhancements, reinforcement strategies address specific functional requirements, such as fire resistance and insulation properties. Incorporating flame-retardant materials and advanced fillers can significantly improve the fire resistance of composites, making them suitable for demanding applications in aerospace and construction [102,103]. In the same way, making polymer nanocomposites with better dielectric properties by strategically adding reinforcements has opened up new ways to store energy and use electronics [15,104].

#### 3.1.2. Matrix Materials and Interfaces

The matrix and interfaces in high-performance advanced composites are pivotal in determining these materials’ overall mechanical, thermal, and durability properties. The matrix is the continuous phase that holds the reinforcement materials together. The interfaces between the matrix and reinforcements make it easier for stress to move, greatly affecting how well the composite works. The choice of matrix material is critical as it affects the composite’s mechanical properties and thermal stability. For instance, thermosetting polymers, such as polyimides, are often selected for their excellent thermal and mechanical properties, making them suitable for aerospace applications [105]. The matrix provides structural integrity and contributes to the composite’s resistance to environmental factors, such as moisture and temperature fluctuations. Studies have shown that the durability of wood-plastic composites is directly related to how the fibers and matrix interact. When the fibers are better encased by the matrix, the performance is better in various environmental conditions [106,107].

**Figure 11:**
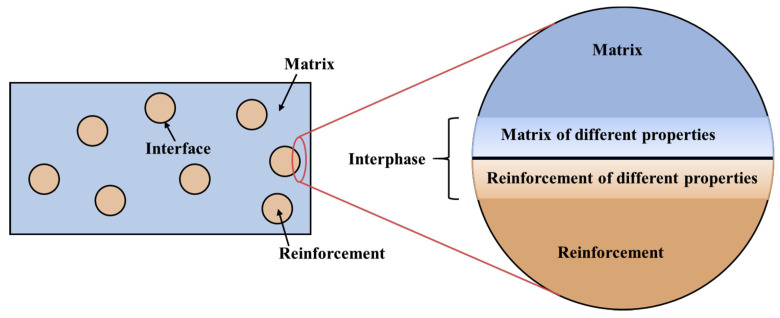
The schematic draw of the interphase in the composite [107].

On a macroscopic scale, the interface is the common boundary between the reinforcement (fibers in the FRP composite) and the polymer matrix. On a microscopic scale, this “boundary” becomes a transition region (called an interphase), which has a finite volume and a zone of extension where the chemical, physical, and mechanical properties change continuously or in stages from the reinforcement to the matrix material. Figure 11 shows a schematic drawing of the interphase in the composite. The interphase is crucial for determining the overall performance and properties of the composite material.

Moreover, the interphase between the matrix and the reinforcement is crucial for effective load transfer. The microstructure of the interface can significantly impact the mechanical properties of the composite. Research indicates that a well-defined interface enhances composites’ mechanical strength and thermal resistance, as it acts as a bridge for stress transfer between the fibers and the matrix [19]. Conversely, poor interfacial bonding can lead to stress concentrations and premature failure, particularly in scenarios involving impact or cyclic loading [108,109]. For example, in carbon fiber-reinforced epoxy composites, the interface’s characteristics directly influence the material’s damage tolerance and overall structural integrity [110]. Advanced composites often incorporate nano-additives or hybrid reinforcements to enhance interfacial properties. Carbon nanotubes or graphene can improve the matrix composites’ load transfer efficiency and thermal conductivity [111]. Studies have shown that changing the interface between these nanomaterials and the matrix can improve performance. For example, changing the interface greatly affected the thermal and mechanical properties [112]. This highlights the importance of selecting the right matrix and optimizing the interface for enhanced performance.

Yet, the processing techniques employed during the fabrication of composites can influence the matrix and interface characteristics. For instance, resin transfer molding (RTM) allows for better control over the matrix’s flow and curing, enhancing the fiber-matrix bonding and reducing void formation [113]. High-pressure microwave processing has also been shown to minimize voids and improve interlayer shear strength, which is critical for aerospace applications [3]. Additionally, high-pressure microwave processing has the potential to reduce processing times compared to traditional methods significantly.

### 3.2. Multifunctional and Smart Composites

Multifunctional and smart composites represent a significant advancement in materials science, offering enhanced capabilities beyond traditional composite materials. These composites are engineered to respond to environmental stimuli, providing self-sensing, actuation, and energy-harvesting functionalities. These are critical in various applications, including aerospace, biomedical devices, and structural health monitoring (SHM) [114,115,116]. Table 2 shows a summary of the design of the multifunctional materials.

One of the key characteristics of multifunctional composites is its ability to integrate multiple functionalities into a single material system. So, adding nanomaterials like graphene and carbon nanotubes (CNTs) to fiber-reinforced polymer (FRP) composites has been shown to improve their mechanical, electrical, and thermal properties without affecting their structural integrity [115,116]. These advancements enable the development of smart composites capable of applications such as EMI shielding, structural health monitoring, and energy storage [115,116]. However, using these nanomaterials effectively promotes problems when scaling up and making the products. Another significant aspect of smart composites is their ability to adapt their properties in response to external stimuli. For example, shape memory alloys (SMAs) embedded within composite matrices can provide active capabilities, allowing the material to change shape or stiffness in response to temperature variations [122,123]. This adaptability is particularly beneficial in applications requiring dynamic responses, such as actuators and sensors. Adding SMAs to regular carbon fiber-reinforced composites has improved the material’s performance, allowing it to self-heal and have adjustable damping properties [24,124].

Moreover, the development of piezoelectric and magnetostrictive materials has further expanded the capabilities of smart composites. These materials can convert mechanical energy into electrical energy and vice versa, making them suitable for applications in energy harvesting and sensing [24,25]. For example, piezoelectric composites can be used in SHM systems to find problems with the structure by observing how the electrical resistance changes when stress is put on them [125]. The ability to self-sense and respond to structural changes is crucial for maintaining the integrity and safety of engineering structures. The design and optimization of multifunctional composites also involve advanced computational techniques. Effective medium theory (EMT) allows for predicting mechanical properties based on the microstructural characteristics of the composite. This theoretical framework aids in understanding how different phases within a composite interact, thereby enabling the design of materials with tailored properties for specific applications [126].

## 4. Challenges in High-Performance Composite Development

Developing high-performance composites has transformed various industries by providing materials with exceptional strength, lightweight properties, and multifunctional capabilities. However, despite their advantages, several challenges must be addressed to unlock the full potential of these composites. Key challenges include mechanical performance limitations, manufacturing scalability, environmental and sustainability concerns, cost-effectiveness, and quality assurance. Each of these areas presents unique obstacles that require innovative solutions and advancements in composite technology.

### 4.1. Mechanical Performance Limitations

Mechanical performance limitations in hybrid composites, particularly those used in aerospace applications, are critical factors that influence their usability and effectiveness. While hybrid composites offer enhanced properties such as improved strength, reduced weight, and better fatigue resistance, they also face several mechanical performance challenges that can hinder their application in demanding environments. One of the primary limitations is the interfacial bonding between the matrix and the reinforcement materials. Poor adhesion can lead to debonding, significantly reducing the composite components’ load transfer efficiency. It has been found that one of the main reasons hybrid titanium composite laminates fail mechanically is that the titanium sheets and fiber-reinforced polymer (FRP) layers come apart. This issue is particularly pronounced in composites with high reinforcement volume fractions, where the stress concentration at the interface can lead to premature failure. To improve the overall performance and dependability of hybrid titanium composite laminates, it is important to understand how the FRP layers fail.

Another significant challenge is the brittleness associated with certain reinforcements. For instance, magnesium hybrid composites that are strengthened with large amounts of ceramic particles like silicon carbide (SiC) and titanium carbide (TiC) often do not bend very easily. This brittleness can lead to catastrophic failure under impact or cyclic loading conditions, which is common in aerospace applications. The inherent properties of the reinforcements, combined with the matrix’s response to stress, can create a strong composite that lacks the necessary toughness for certain applications. Additionally, the processing techniques used to fabricate hybrid composites can impose limitations on their mechanical performance. For instance, methods such as friction stir processing can introduce defects or inconsistencies in the microstructure, which can adversely affect the mechanical properties of the final product. Variations in processing parameters can lead to differences in grain size, phase distribution, and reinforcement dispersion, ultimately impacting the overall performance of the composite.

Additionally, the mechanical properties of hybrid composites can be sensitive to environmental factors such as temperature and humidity. For example, studies have shown that the mechanical performance of magnesium-based composites can degrade significantly at elevated temperatures, which is a critical consideration for aerospace applications where components are often subjected to extreme thermal conditions. The thermal stability of the matrix and the reinforcements must be carefully considered to ensure reliable service performance. Finally, the complexity of hybrid composites, which often involve multiple materials and interfaces, can complicate predicting their mechanical behavior. The interactions between different phases can lead to unexpected failure modes, making it challenging to design composites that meet specific performance criteria. Understanding the synergistic effects of various reinforcements and the resulting mechanical properties is essential for optimizing hybrid composites for aerospace applications.

In conclusion, while hybrid composites present significant advantages for aerospace applications, their mechanical performance limitations must be addressed through careful material selection, processing techniques, and design considerations. Ongoing research into improving interfacial bonding, enhancing ductility, and understanding the effects of environmental conditions will be crucial for advancing the application of hybrid composites in the aerospace industry.

### 4.2. Manufacturing and Scalability Issues

Manufacturing and scalability issues in high-performance composites are critical challenges that impact their adoption across various industries, particularly aerospace applications. While these composites offer significant advantages, such as enhanced mechanical properties and reduced weight, their production processes often face limitations that hinder large-scale implementation. One of the primary manufacturing challenges is achieving uniform dispersion of reinforcements within the composite matrix. Incorporating nanomaterials, such as carbon nanotubes or graphene, into polymer or metal matrices can lead to difficulty maintaining consistent distribution throughout the material. Traditional blending methods, such as ball milling, often struggle to achieve the necessary homogeneity, especially at high filler concentrations. This non-uniformity can result in localized weaknesses, adversely affecting the overall mechanical performance of the composite.

Moreover, the manufacturing processes can be complex and require precise control over various parameters. Techniques such as automated fiber placement (AFP) and additive manufacturing (AM) have emerged as advanced methods for producing high-performance composites. However, these methods come with challenges, including the need for specialized equipment and expertise, which can complicate production scaling. The optimization of processing parameters, such as temperature, pressure, and curing times, is essential to ensure the desired properties of the final product, but this can be a time-consuming and resource-intensive process. Another significant issue is the economic viability of producing high-performance composites at scale. The costs associated with raw materials, advanced manufacturing techniques, and quality control can make these composites less competitive than traditional ones. For instance, the production of thermoset polymers, often used in high-performance applications, has seen annual outputs exceeding 60 million tons. Yet, the demand for lightweight materials continues to drive the need for more efficient and cost-effective manufacturing solutions.

Additionally, the environmental impact of composite manufacturing processes is becoming increasingly important. High-performance composites often involve using hazardous materials and energy-intensive processes, raising concerns about sustainability and environmental compliance. Efforts to develop greener manufacturing techniques and recycling methods for composite materials are ongoing, but scalability remains a challenge. Finally, integrating advanced manufacturing technologies, such as continuous processing and high-throughput synthesis, is essential for addressing scalability issues. Recent advancements in the scalable production of boron nitride nanosheets have demonstrated the potential for creating high-performance composites with improved mechanical and thermal properties. However, transitioning from laboratory-scale production to full-scale manufacturing requires careful consideration of process scalability and maintaining quality control throughout production. In conclusion, while high-performance composites offer significant advantages for various applications, their manufacturing and scalability issues present challenges that must be addressed. High-performance composites in aerospace and other fields will succeed if they achieve even reinforcement distribution, find the best processing parameters, make the materials cost-effective, minimize environmental impact, and efficiently scale up advanced manufacturing techniques.

### 4.3. Environmental and Sustainability Concerns

Environmental and sustainability concerns regarding high-performance composites are increasingly significant as industries strive to reduce their ecological footprint while maintaining performance standards. High-performance composites, often used in aerospace, automotive, and construction applications, present unique challenges and opportunities related to their environmental impact throughout their life cycle. One of the primary concerns is the environmental impact associated with the production of synthetic composites. Most high-performance composites are made from petroleum-based materials, contributing to greenhouse gas emissions and resource depletion during extraction and processing. The manufacturing processes for these composites are often energy-intensive, leading to a substantial carbon footprint. For instance, the life cycle assessment (LCA) of synthetic composites used in aviation has indicated that while they offer performance benefits, their production contributes significantly to atmospheric emissions, particularly due to the energy required for processing.

In contrast, natural fiber-reinforced composites (NFRCs) have emerged as a more sustainable alternative. Studies have shown that NFRCs, which utilize hemp, flax, or jute materials, generally have lower environmental impacts than their synthetic counterparts. This is primarily due to the lower energy requirements for cultivating and processing natural fibers and their biodegradability at the end of their life cycle [127]. However, the use of natural fibers is not without its challenges. While they offer environmental benefits, natural fibers can exhibit variability in mechanical properties due to factors such as growth conditions and processing methods. This variability can complicate the design and manufacturing processes, potentially limiting the performance of the final composite.

Also, integrating natural fibers into high-performance composites often requires careful consideration of the matrix material to ensure compatibility and optimal performance [124]. Recycling and end-of-life management of high-performance composites also pose significant environmental challenges. Synthetic composites are often difficult to recycle due to the strong bonding between the matrix and the reinforcement, which complicates the separation of materials for recycling. This issue raises concerns about the accumulation of composite waste in landfills and the associated environmental impacts. In contrast, natural fiber composites tend to be more amenable to recycling and composting, aligning better with sustainability goals [108]. However, the infrastructure for recycling natural fiber composites is still underdeveloped, which limits their potential environmental benefits.

Moreover, the development of bio-based composites is gaining traction as a sustainable alternative. These composites utilize renewable resources, such as plant fibers and bio-based resins, which can significantly reduce reliance on fossil fuels and lower carbon emissions during production. Research has shown that bio-based composites can achieve comparable mechanical properties to traditional composites while offering enhanced sustainability [125]. However, the scalability of bio-based composite production remains a challenge, as it requires the establishment of new supply chains and processing technologies.

## 5. Future Directions in Multifunctional Composite Design

The future directions in multifunctional composite design are poised to significantly influence various industries, particularly the aerospace, automotive, and construction industries. As the demand for materials that can perform multiple functions simultaneously increases, researchers and engineers focus on innovative approaches to enhance these composites’ performance, sustainability, and applicability. One promising avenue is the integration of advanced nanomaterials into composite matrices. Incorporating CNTs, graphene, and metal–organic frameworks (MOFs) can significantly enhance composites’ mechanical, thermal, and electrical properties. For instance, recent studies have demonstrated that graphene-based natural fiber composites exhibit excellent mechanical and thermal properties, making them suitable for multifunctional applications such as structural health monitoring and EMI shielding [110]. The ability to tailor the properties of these composites through nanomaterial reinforcement opens new possibilities for their use in high-performance applications.

Moreover, the development of bio-based and sustainable composites is gaining traction as environmental concerns become more prominent. Using natural fibers, such as jute and hemp, combined with bio-based resins, can lead to composites that are high-performing and environmentally friendly [23]. These materials can provide excellent mechanical properties while reducing reliance on fossil fuels, aligning with the growing emphasis on sustainability in material design. The exploration of multifunctional composites incorporating bio-based materials is expected to expand, particularly in sectors where eco-friendliness is a priority.

Another significant trend is the advancement of smart composites that can respond to external stimuli. Research into materials that exhibit self-healing properties, shape memory effects, or those that can change their properties in response to environmental conditions is on the rise. For example, developing multifunctional composites that integrate phase change materials (PCMs) for thermal management can enhance energy efficiency in buildings and vehicles [126,127]. These materials can absorb, store, and release thermal energy, improving performance in various applications. The integration of multifunctional capabilities into textiles is another exciting direction. Incorporating sensors and electronic components into fabrics can lead to developing smart textiles that monitor health and environmental conditions or even provide energy-harvesting capabilities [128]. The potential for fiber-integrated materials to serve multiple functions, such as structural support and sensing, represents a significant advancement in multifunctional composites.

Furthermore, using artificial intelligence (AI) and machine learning in designing and optimizing multifunctional composites is emerging as a transformative approach. AI can facilitate the identification of optimal material combinations and processing conditions, leading to enhanced performance and reduced manufacturing costs [129]. Integrating digital technologies into material design processes is expected to accelerate the development of advanced multifunctional composites.

In conclusion, the future of multifunctional composite design is characterized by integrating advanced materials, sustainable practices, smart functionalities, and digital technologies. As researchers continue to explore these avenues, the potential for multifunctional composites to revolutionize various industries becomes increasingly apparent. The ongoing innovations in material science will enhance composites’ performance and contribute to a more sustainable and efficient future.

## 6. Conclusions

This review of high-performance advanced composites highlights the transformative potential of these materials across various sectors, especially in applications requiring a balance between lightweight properties, high strength, and multifunctionality. Key findings emphasize the advantages of fiber-reinforced composites, particularly those incorporating carbon, glass, aramid, and nanofibers. These materials offer unique mechanical, thermal, and environmental properties, enabling applications ranging the from aerospace and automotive industries to energy and defense. Choosing the right matrix materials, like metals, ceramics, and polymers, and using advanced interface engineering methods is key to obtaining the best performance out of composites and making them last longer, even in harsh conditions. Significant advancements have been achieved in composite manufacturing techniques, particularly with automated and additive manufacturing methods. These methods enhance manufacturing precision, reduce waste, and enable the creation of complex, highly customized structures.

Multifunctional composites are also a big step forward because they combine structural properties with the ability to store energy and sense things. They are in line with the trend toward smart, integrated material systems. Despite these advances, challenges still need to be addressed, particularly regarding recyclability, scalability, cost, and the need for robust quality assurance standards. Developing sustainable and bio-based composites and efficient recycling solutions is necessary to mitigate environmental impact and ensure the long-term viability of composite technologies. Continued exploration of hybrid and nanocomposites is recommended to achieve multifunctionality without compromising structural integrity. Additionally, research into sustainable raw materials and manufacturing processes is crucial to further reducing the environmental footprint of composites.

## Figures and Tables

**Table 1:** Overview of hybrid composite design and the application and advantages of these materials.

Design	Advantages	Applications	References
Carbon/Glass Fiber Hybrid	Enhanced mechanical properties, improved impact resistance, and reduced weight.	Aerospace, automotive, and structural components.	[61,62]
Jute/Carbon Hybrid	Cost-effective, lightweight, and sustainable with good mechanical performance.	Automotive, building materials, and medical applications	[63,64]
Basalt/Glass Fiber Hybrid	High thermal stability and resistance to environmental degradation	Construction and civil engineering	[65]
Aramid/Carbon Fiber Hybrid	Superior toughness and impact resistance, suitable for high-stress applications	Military, aerospace, and protective gear.	[66]
Natural/Synthetic Fiber Hybrid	Improved mechanical properties and sustainability, lower environmental impact.	Biocomposites in construction and automotive sectors.	[67]
Kevlar/Glass Fiber Hybrid	Balanced strength and flexibility, suitable for protective applications.	Personal protective equipment and sports gear.	[68]
Sandwich Hybrid Composites	Lightweight with high strength-to-weight ratio, excellent thermal insulation.	Aerospace, automotive, and building materials.	[69]

**Table 2:** Design of the multifunctional materials.

Configuration	Design	Reference
Gradient materials	These materials exhibit a gradual change in composition and properties across their volume, enhancing performance in applications such as aerospace and automotive components	[117]
Sandwich structures	Composed of two outer layers and a core material, these structures provide high strength-to-weight ratios and are commonly used in thermal insulation and aerospace applications.	[118]
Thermal interface materials	These materials combine polymers with conductive fillers to reduce thermal resistance at interfaces, crucial for electronic devices.	[119]
Functional graded materials	These materials have a continuous variation in composition and structure, improving wear resistance and thermal properties, widely used in aerospace and automotive sectors.	[120]
Multi-functional structures	Hybrid designs that combine various materials to meet multiple functional requirements, such as lightweight and high strength.	[121]
